# Chilling Stress—The Key Predisposing Factor for Causing *Alternaria alternata* Infection and Leading to Cotton (*Gossypium hirsutum* L.) Leaf Senescence

**DOI:** 10.1371/journal.pone.0036126

**Published:** 2012-04-27

**Authors:** Jingqing Zhao, Sha Li, Tengfei Jiang, Zhi Liu, Wenwei Zhang, Guiliang Jian, Fangjun Qi

**Affiliations:** State Key Laboratory for Biology of Plant Diseases and Insect Pests, Institute of Plant Protection, Chinese Academy of Agricultural Sciences, Beijing, People's Republic of China; Max Planck Institute for Chemical Ecology, Germany

## Abstract

Leaf senescence plays a vital role in nutrient recycling and overall capacity to assimilate carbon dioxide. Cotton premature leaf senescence, often accompanied with unexpected short-term low temperature, has been occurring with an increasing frequency in many cotton-growing areas and causes serious reduction in yield and quality of cotton. The key factors for causing and promoting cotton premature leaf senescence are still unclear. In this case, the relationship between the pre-chilling stress and *Alternaria alternata* infection for causing cotton leaf senescence was investigated under precisely controlled laboratory conditions with four to five leaves stage cotton plants. The results showed short-term chilling stress could cause a certain degree of physiological impairment to cotton leaves, which could be recovered to normal levels in 2–4 days when the chilling stresses were removed. When these chilling stress injured leaves were further inoculated with *A. alternata*, the pronounced appearance and development of leaf spot disease, and eventually the pronounced symptoms of leaf senescence, occurred on these cotton leaves. The onset of cotton leaf senescence at this condition was also reflected in various physiological indexes such as irreversible increase in malondialdehyde (MDA) content and electrolyte leakage, irreversible decrease in soluble protein content and chlorophyll content, and irreversible damage in leaves' photosynthesis ability. The presented results demonstrated that chilling stress acted as the key predisposing factor for causing *A. alternata* infection and leading to cotton leaf senescence. It could be expected that the understanding of the key factors causing and promoting cotton leaf senescence would be helpful for taking appropriate management steps to prevent cotton premature leaf senescence.

## Introduction

Senescence is a natural and complex process in all biological organisms. In plant, leaf senescence acts as an important step in plant life cycle and an integral part of plant growth and development. Leaf senescence, typical and visual symptom tissue and organ senescence for most of plants, showed yellowing, desiccation, and eventual abscission in leaves [1], usually occurs in an age-dependent manner, plays a vital role in nutrient recycling [2], and also acts as a determinant process for yield production in many crops [3]. Thus, although leaf senescence is a deleterious process for the sake of the leaf organ, it can be seen as an altruistic process: it critically contributes to the fitness of whole plants by ensuring optimal production of offspring and better survival of plants in their given temporal and spatial niches. Leaf senescence is thus an evolutionarily selected developmental process and comprises an important phase in the plant life cycle [4]–[6]. Chlorophyll loss had been widely used for the characterization of the leaf senescence syndrome [7]. Other changes like irreversible protein degradation and photosynthetic impairment [8], dramatic irreversible increase in lipid peroxidation and membrane electrolyte leakage were also used as the physiological indexes in the characterization of plant leaf senescence syndrome [9].

In agricultural aspects, leaf senescence too early (premature senescence) or too late (late maturity) may have great influence on yield production for annual crops. Late maturity would interfere with nutrient remobilization, thereby compromising photosynthetic activity in young leaves and reproductive success. By contrast, premature leaf senescence would reduce the plant's overall capacity to assimilate carbon dioxide and stunt plant growth [10].

Cotton (*Gossypium hirsutum* L.), the most important plant fiber production crop, is a perennial, indeterminate plant that is cultured as an annual in agronomic production systems. However, cotton premature leaf senescence has been occurring with an increasing frequency in many cotton-growing countries, especially in China, and causes serious reduction in yield and quality of cotton [11]. Early leaf senescence results in reduced lint yield and poor fiber properties, thus constituting an important constraint to cotton yield and quality [12]. Lint yield loss can be as high as 20% in the USA [13]. In China, a significant yields reduction of 20–50% was recorded in severe premature leaf senescence occurred years [14]. Premature leaf senescence has developed to be one of the important restricted barriers for cotton production in China in recent years.

The key factors for causing and promoting cotton premature leaf senescence are still unclear. As reported previously, cotton premature leaf senescence may occur under the complex interaction between developmental age and environment factors such as nutrient deficiency [12], drought [15], [16], elevated carbon dioxide [17], and salinity [18], or internal factors such as phytohormones [19], [20]. As reported in Australia, large boll load and potassium deficiency are likely to constitute the most important factors for causing cotton premature leaf senescence [21], [22].

In China, it was reported that the appearance of severe cotton leaf senescence in major cotton growing area often accompanied with unexpected short-term low temperature that occurred in the late cotton growing season, especially in early-to-mid of August. For example, it was recorded that in northern Xinjiang, the temperature was below 19°C for consecutive 3 days in August, 2004, the minimum temperature was only 8.4°C, about 1–2 week after low temperature weather condition, premature senescence symptoms become so severe that eventually the whole plant was defolicated over the cotton growing fields. During this natural calamity, cotton premature senescence occurred across much of the cotton production region and the production dropped by 30–40% [23]. However, it had been proved that cotton plant could recover from adverse temperature conditions, such as freezing, when temperatures returned to optimal [24]. So the low temperature could not act as the single factor for causing cotton premature leaf senescence. It should further investigate the other possible factors which may interact with the cold cloudy weather for causing the occurrence of cotton premature leaf senescence. Our previous field investigation showed that almost each cotton senescent leaf, frequently appeared after a period of consecutive low temperature days, was always accompanied with a large amount of leaf spot lesions. The main isolated pathogens from these disease lesions were identified to be *Alternaria alternata* [25].

As is known, Alternaria leaf spot of cotton occurs in most cotton growing countries [26]. At least two pathogens, *A. macrospora* and *A. alternata* are considered to be the main causal agents [27]. There was no report of the impact of this disease on cotton production in China, but a yield loss of 37% was reported in India [28] and of 25% in Israel [29]. Although most upland cultivars are not normally susceptible to severe infection by these two pathogens, they may become predisposed to infection by several stress factors such as nutrient imbalance and water stress [30], or nematode attack [31]. In general, infection of upland cultivars by *Alternaria* spp. was more severe following periods of adverse growing conditions [32], and the occurrence of Alternaria disease was often accompanied with cotton leaf senescence [33]. Whether chilling stress acts as a predisposing factor for trigging *A. alternata* infection on cotton leaves and promoting cotton leaf senescence is still unknown.

In this study, the investigation was conducted to reveal the relationship of chilling stress, *A. alternata* infection and cotton leaf senescence. Further, the chilling stress acted as the key predisposing factor for causing *A. alternata* infection and leading to cotton (*Gossypium hirsutum* L.) leaf senescence was firstly reported.

## Results

### Influences of chilling stress pre-treatments on *A. alternata* infection and disease development

**Figure 1:**
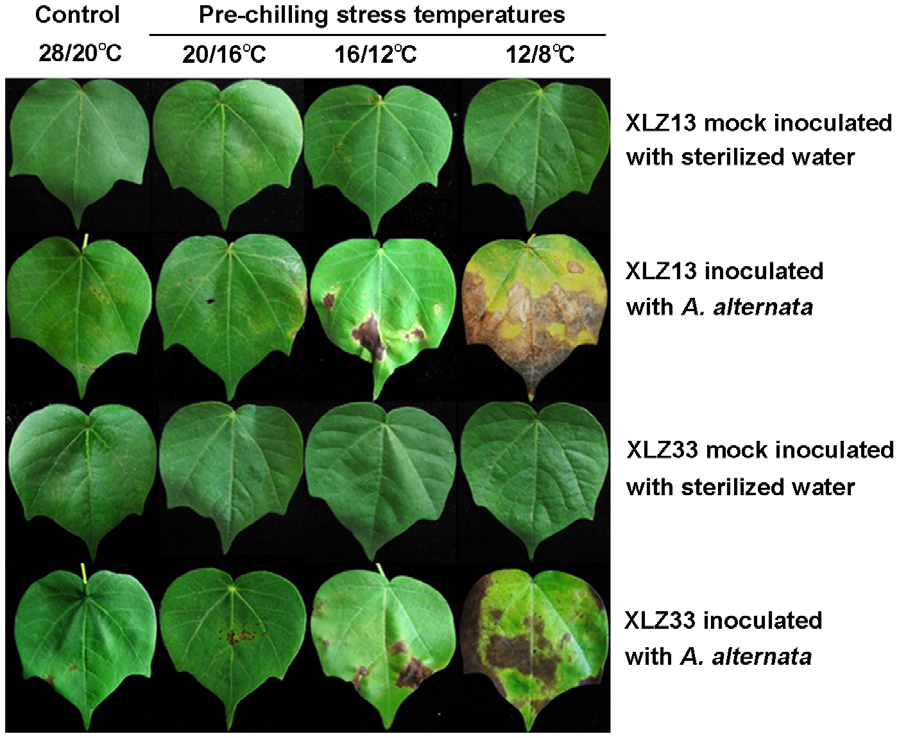
Appearance of Alternaria disease on cotton leaves pre-treated by chilling stress with various low temperatures. Chilling stress pre-treatments were performed with various low temperatures for 3 days. Then the pre- treated cotton leaves were inoculated with 1.2×10^4^ conidial/mL inoculum suspension of *A. alternata* isolate A1 by slightly brushing method and returned to grow at optimal temperature of 28/20°C. The mock inoculations were performed with sterilized water. Presented pictures photographed at 15 days after inoculation.

The results showed only small necrotic lesions appeared at 7 days after inoculation, but hardly expanded and developed on the inoculated leaves of control plants maintained at optimal temperature (28/20°C), suggesting that leaves free of chilling stress pre-treatment were resistant to *A. alternata* infection at a certain degree (Figure 1, control leaves for XLZ13 and XLZ33 inoculated with *A. alternata*).

While these chilling stress pre-treated leaves showed more susceptible to *A. alternata* in two aspects: firstly, the appearances of disease lesions were earlier in chilling stress pre-treated leaves than that of control (at 6–7 days, 5–6 days and 3–4 days for 20/16°C, 16/12°C, 12/8°C chilling stress pre-treatments, respectively. Table 1); secondly, the disease lesions were continuously developed and expanded in chilling stress pre-treated leaves, which showed more and larger disease lesions in these leaves after inoculation in a time-dependent manner, as the pre-treated temperature was getting lower (Figure 1).

**Table 1:** Influences of chilling stress pre-treatments performed with various low temperatures on occurrence and development of Alternaria disease.

Pre-chilling stress temperatures	Initial days to appear lesion after inoculation (d)		Disease index at 15 days after inoculation	
	XLZ13	XLZ33	XLZ13	XLZ33
28/20°C (Control)	7	7	9.0 a a	9.0 a a
20/16°C	6	7	17.0 b a	15.0 b a
16/12°C	5	5.5	49.0 c a	43.0 c b
12/8°C	3	4	85.0 d a	80.0 d b

Cotton seedlings were pre-treated with chilling stress for 3 days, then inoculated with *A. alternata* isolate A1, and removed to grow at optimal temperature of 28/20°C; Different letters behind the disease index indicate significant difference with the *P* value of 0.05. The first letters indicate the comparison among different temperatures of chilling stress pre-treatment and the second letters indicate the comparison between XLZ13 and XLZ33.

The disease index also indicated that chilling stress pre-treatments could promote the development of leaf spot disease. As shown in Table 1, the disease index detected at 15 days after inoculation was only 9.0 for control leaves of XLZ13, while the detected disease index were significantly higher in all low temperature pre-treatments with the values of 17.0, 49.0, 85.0 for 20/16°C, 16/12°C, 12/8°C, respectively.

It was also noticed that chilling stress pre-treatments showed similar promoting effects on leaf spot disease development for XLZ33. However, the differences in process and severity for the leaf spot disease could be obviously distinguished between XLZ13 and XLZ33. Compared to XLZ13, the appearances of disease lesion were 0.5 and 1 day later, and the detected disease index for XLZ33 was significantly lower in 16/12°C, 12/8°C chilling stress pre-treatments, respectively (Table 1). The differences reflected that the leaf senescence resistant cultivars may be also resistant to leaf spot disease at a certain degree.

**Figure 2:**
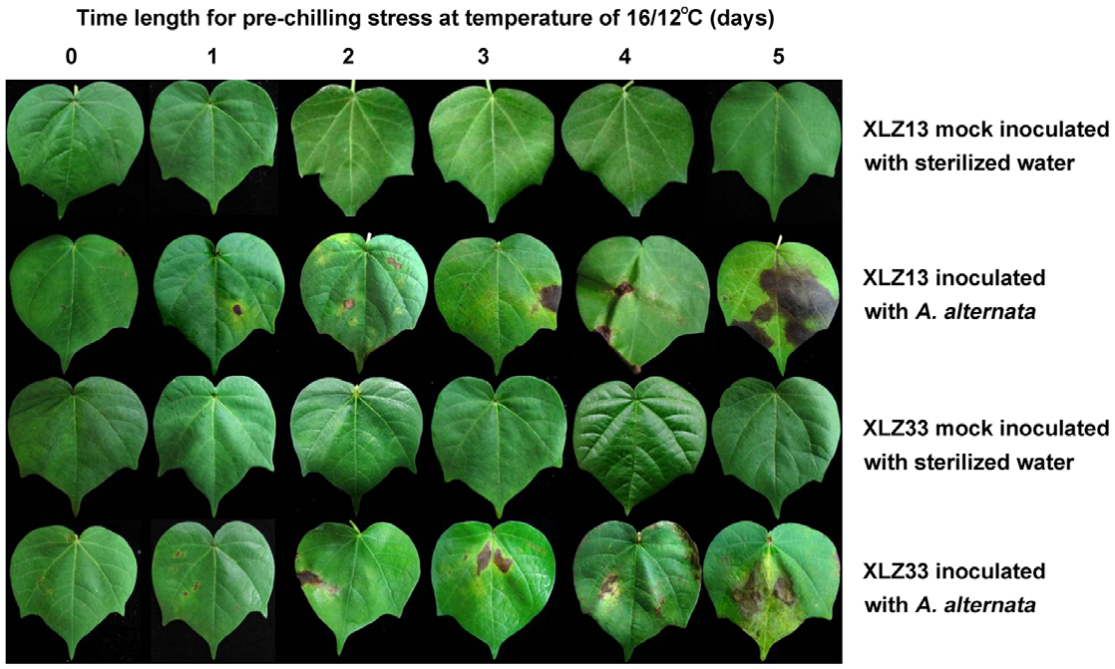
Appearance of Alternaria disease on cotton leaves pre-treated by various durations of chilling stress at 16/12°C. Various durations of chilling stress pre-treatments were performed at 16/12°C. Then the pre-treated cotton leaves were inoculated with 1.2×10^4^ conidial/mL inoculum suspension of *A. alternata* isolate A1 by slightly brushing method and returned to grow at optimal temperature of 28/20°C. The mock inoculations were performed with sterilized water. Presented pictures photographed at 15 days after inoculation.

In order to further investigate the influence of chilling stress pre-treatment on the *A. alternata* infection and leaf spot disease development, various durations of chilling stress pre-treatments at 16/12°C were performed. As Table 2 showed, with the prolonged pre-exposure to the low temperature of 16/12°C, the less time for the appearance of disease lesions, and the higher increase in disease index values at 15 days after inoculation were recorded. For example, in the case of XLZ13 and XLZ33 leaves pre-treated with the low temperature of 16/12°C for 5 days, the appearances of disease lesions were only 3 days and 4 days, the disease indexes were 68.0 and 63.0, respectively, whereas in control leaves of XLZ13 and XLZ33 (untreated with chilling stress), the lesions were observed until 7 day after inoculation and the detected disease indexes were only 9.0 (Table 2). As expected, the appearance of more and larger disease lesions in the leaves of both cultivars with prolonged pre-exposure to the low temperature of 16/12°C were observed (Figure 2). However, the differences in the leaf spot disease development and severity could also be distinguished between XLZ13 and XLZ33 in these cases. Compared to XLZ13, the appearances of disease lesions for XLZ33 were 0.5 and 1 day later in the leaves pre-treated with chilling stress at 16/12°C for 3 and 5 days, and the detected disease index for XLZ33 were significantly lower in the leaves pre-treated with chilling stress at 16/12°C for 3, 4 and 5 days, respectively (Table 2).

**Table 2:** Influences of chilling stress pre-treatments performed with various durations of chilling stress at 16/12°C on occurrence and development of Alternaria disease.

Various durations of chilling stress at 16/12°C (d)	Initial days to appear lesion after inoculation (d)		Disease index at 15 days after inoculation	
	XLZ13	XLZ33	XLZ13	XLZ33
0	7	7	9.0 a a	9.0 a a
1	6	7	15.0 b a	13.0 a a
2	6	6	20.0 c a	18.0 b a
3	5	5.5	54.0 d a	43.0 c b
4	4	4	57.0 d a	52.0 d b
5	3	4	68.0 e a	63.0 e b

Cotton seedlings were pre-treated with various duration of chilling stress at 16/12°C, then inoculated with *A. alternata* isolate A1, and removed to grow at optimal temperature of 28/20°C; Different letters behind the disease index indicate significant difference with the *P* value of 0.05. The first letters indicate the comparison among various duration of chilling stress at 16/12°C and the second letters indicate the comparison between XLZ13 and XLZ33.

In conclusion, it had been proved that chilling stress pre-treatments could promote *A. alternata* infection and cause leaf spot disease in chilling stress pre-treatments conducted with various low temperatures for 3 days or various durations at 16/12°C. These results indicated that chilling stress pre-treatment actually acted as a key predisposing factor for causing *A. alternata* infection and leading to the appearance of leaf spot disease. The results also indicated that the leaf senescence resistant cultivar XLZ33 was also more resistant to *A. alternata* under chilling stress pre-treatment conditions.

### Changes in malondialdehyde (MDA) content and electrolyte leakage during Alternaria disease development promoted by chilling stress pre-treatment

**Figure 3:**
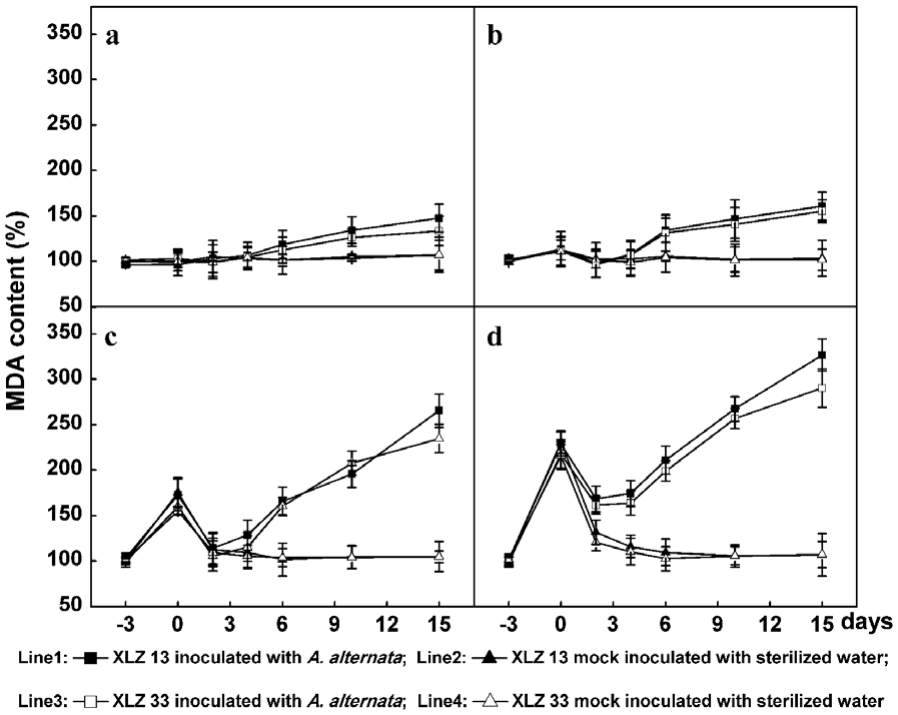
Changes in MDA content during Alternaria disease development promoted by chilling stress pre-treatment. a: the control cotton plants sustained growing at optimal temperature of 28/20°C. b, c, d: Cotton plants performed with chilling stress pre-treatments at 20/16°C, 16/12°C, 12/8°C for 3 days respectively, then inoculated with 1.2×10^4^ conidial/mL inoculum suspension of *A. alternata* isolate A1, and returned to grow at 28/20°C. All collected data (mean ± standard deviation SD with 6 replicates) were presented as relative values to the malondialdehyde contents at −3 d (100% MDA content = 22.4 nmol/gFW for XLZ13 and 21.8 nmol/gFW for XLZ33 leaves, respectively). The chilling stress pre-treatment period was indicated by time points from −3 to 0, and the period after inoculation was indicated by time points from 0 to 15.

**Figure 4:**
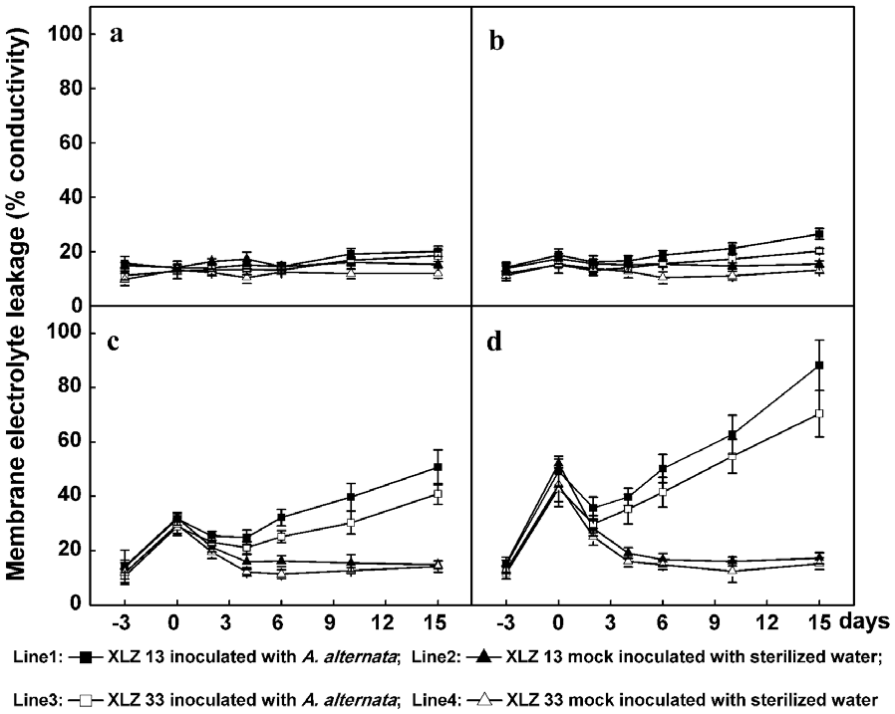
Changes in electrolyte leakage during Alternaria disease development promoted by chilling stress pre-treatment. a: the control cotton plants sustained growing at optimal temperature of 28/20°C. b,c,d: Cotton plants performed with chilling stress pre-treatments at 20/16°C, 16/12°C, 12/8°C for 3 days respectively, then inoculated with 1.2×10^4^ conidial/mL inoculum suspension of *A. alternata* isolate A1, and returned to grow at 28/20°C. All collected data (mean ± standard deviation SD with 6 replicates) were presented as relative values of membrane electrolyte leakage. The chilling stress pre-treatment period was indicated by time points from −3 to 0, and the period after inoculation was indicated by time points from 0 to 15.

The lipid peroxidation products MDA and electrolyte leakage were routinely used as indicators of functionality and integrity of cell membrane [34]. Compared to control leaves, both MDA content and electrolyte leakage were increased in leaves during chilling stress pre-treatments, with the greatest increase in the leaves chilled at the lowest temperature (Figure 3c, d; Figure 4c, d). However, the increased MDA and electrolyte leakage gradually recovered to normal levels in 2–4 days when the chilling stresses were discarded and cotton plants were maintained to grow at optimal temperature again (Figure 3c, d: line 2, 4; Figure 4c, d: line 2, 4). These results indicated that short-time chilling stress could only cause temporary membrane impairment which could be repaired reversibly. In other words, the results suggested it might be impossible for short-time chilling stress to cause permanent cell membrane damages and eventually lead to cotton leaf cell death.

The cell membrane repairing processes were completely different when the leaves were further inoculated with *A. alternata* after chilling stress pre-treatment. Cell membrane integrity and function could be recovered temporarily, not completely, during the first 2 days after inoculation, as indicated by a slight decrease in both increased MDA and electrolyte leakage induced by chilling stress pre-treatment. Since then, these repairing processes were definitely reversed as reflected by persistent increase in MDA content and electrolyte leakage (Figure 3c, d: line 1, 3; Figure 4c, d: line 1, 3) accompanied with Alternaria disease development. Among the different low temperature pre-treatments evaluated, MDA and membrane ion leakage increase were more dramatic on the leaves with severe infection occurred in the 12/8°C pre-treated leaves, as shown in Figure 4d: line 1, 3, the relative values of membrane electrolyte leakage were increased to 70.4%, 88.2% and MDA contents were increased to 290.4%, 326.6% for 12/8°C pre-treated XLZ33, XLZ13 at 15 days after inoculation, respectively, suggesting that the integrity and function of cell membrane had been damaged completely and irreversibly.

### Changes in chlorophyll and soluble protein content during Alternaria disease development promoted by chilling stress pre-treatment

**Figure 5:**
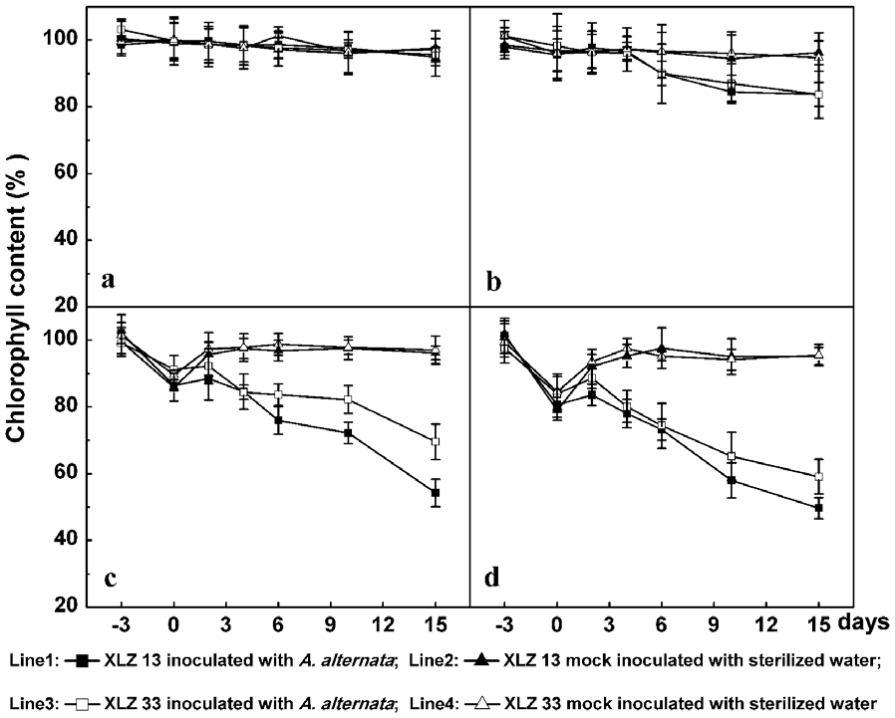
Changes in chlorophyll contents during Alternaria disease development promoted by chilling stress pre-treatment. a: the control cotton plants sustained growing at optimal temperature of 28/20°C. b, c, d: Cotton plants performed with chilling stress pre-treatments at 20/16°C, 16/12°C, 12/8°C for 3 days respectively, then inoculated with 1.2×10^4^ conidial/mL inoculum suspension of *A. alternata* isolate A1, and returned to grow at 28/20°C. All collected data (mean ± standard deviation SD with 6 replicates) were presented as relative values to the chlorophyll contents (SPAD unit) at −3 d (100% chlorophyll content = 41.5 for XLZ13 and 44.0 for XLZ33 leaves, respectively). The chilling stress pre-treatment period was indicated by time points from −3 to 0, and the period after inoculation was indicated by time points from 0 to 15.

Chlorophyll is an extremely important and critical biomolecule in photosynthesis with function of light absorbance and light energy transformation. Results showed that leaf chlorophyll contents for XLZ13 and XLZ33 were almost maintained quite stable when grown at optimal temperature (28/20°C) (Figure 5a). Under this growth condition, even inoculating with *A. alternata* could not cause pronounced changes in leaf chlorophyll content which were almost kept at a relative stable level (Figure 5a), and this corresponded to few lesions development in this case mentioned above. Pre-treatment with relatively low temperature of 20/16°C decreased leaf chlorophyll content slightly for XLZ13 and XLZ33 (Figure 5b: line 2, 4), a further decrease in chlorophyll contents were not significant despite of the chloroses/necroses development after inoculation (Figure 5b: line 1, 3). Pre-treatment with much lower temperatures of 16/12°C and 12/8°C decreased chlorophyll contents significantly (Figure 5c, d: 0 day point showed). Figure 5d showed chlorophyll contents for XLZ13 and XLZ33 were significantly reduced by 20–22% and 13–15% by 3 days chilling stress pre-treatment at low temperature of 12/8°C, respectively. However, the decreased leaf chlorophyll contents for XLZ13 and XLZ33 could be recovered completely over 2–4 days growing at optimal temperature (Figure 5c, d: line 2, 4). Figure 5d (line 1, 3) indicated that chlorophyll levels did drop considerably after slight increase following the inoculation, the contents of chlorophyll were decreased to 49.7%, 59.1% for XLZ13 and XLZ33, measured at 15 days after inoculation, respectively. The data were consistent with the notion that leaves pre-treated with the lowest temperature showed pronounced disease symptoms, including the highest levels of chlorotic lesions and visible yellowing. Thus, on the base of obtained results it may be concluded that the irreversible decline in the leaf chlorophyll content was accounted for by the direct influence of the faster and greater development of the Alternaria disease in the chilling stress pre-treated leaves.

**Figure 6:**
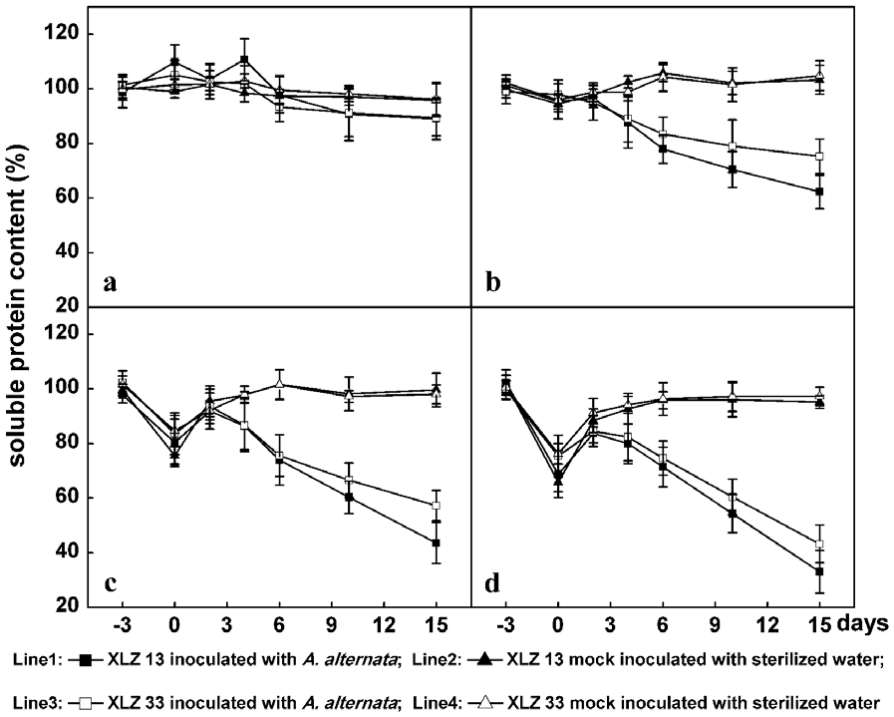
Changes in soluble protein contents during Alternaria disease development promoted by chilling stress pre-treatment. a: the control cotton plants sustained growing at optimal temperature of 28/20°C. b, c, d: Cotton plants performed with chilling stress pre-treatments at 20/16°C, 16/12°C, 12/8°C for 3 days respectively, then inoculated with 1.2×10^4^ conidial/mL inoculum suspension of *A. alternata* isolate A1, and returned to grow at 28/20°C. All collected data (mean ± standard deviation SD with 6 replicates) were presented as relative values to the soluble protein content at −3 d (100% soluble protein content = 24.8 mg/gFW for XLZ13 and 27.5 mg/gFW for XLZ33 leaves, respectively). The chilling stress pre-treatment period was indicated by time points from −3 to 0, and the period after inoculation was indicated by time points from 0 to 15.

Soluble proteins were basic and vital biomolecules involved in various biological metabolisms. As shown in Figure 6, the changes in soluble protein contents showed approximately similar character with the changes in chlorophyll contents. At optimal growth temperature of 28/20°C, soluble protein contents were kept at a relatively stable level on the leaves inoculated or un-inoculated with *A. alternata* (Figure 6a). While, as Figure 6c, d showed, pre-chilling stress treatments could cause temporary decrease in soluble protein contents of leaves, which could be recovered reversibly after the cotton plants returned to optimal temperature again. When chilling stress pre-treated leaves were further inoculated with *A. alternata*, soluble protein contents were increased upon 2 days, following by irreversible decrease accompanied with the occurrence and development of cotton leaf spot disease mentioned above. *A. alternata* infection for 15 days retained 89.0%, 62.2%, 43.6%, 33.0% of their initial soluble protein in 28/20°C, 20/16°C, 16/12°C, 12/8°C pre-treated leaves, respectively. The soluble protein content of XLZ33 leaves dropped to 89.3%, 75.2%, 57.21%, 43.2% in response to treatment with 28/20°C, 20/16°C, 16/12°C, 12/8°C, respectively, after 15 days after inoculation (Figure 6: line 1, 3). The results indicated that soluble protein contents showed to be decreased irreversibly accompanied with the occurrence and development of cotton leaf spot disease in chilling stress pre-treated and further inoculated leaves.

### Changes in Fv/Fm ratio during Alternaria disease development process promoted by chilling stress pre-treatment

**Figure 7:**
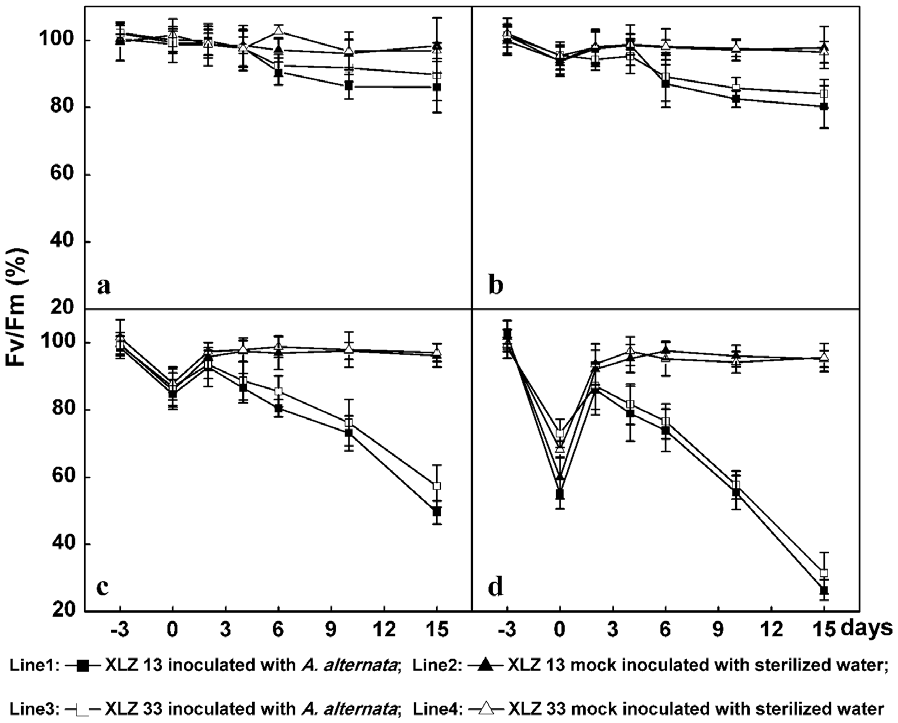
Changes in Fv/Fm ratio during Alternaria disease development promoted by chilling stress pre-treatment. a: the control cotton plants sustained growing at optimal temperature of 28/20°C. b, c, d: Cotton plants performed with chilling stress pre-treatments at 20/16°C, 16/12°C, 12/8°C for 3 days respectively, then inoculated with 1.2×10^4^ conidial/mL inoculum suspension of *A. alternata* isolate A1, and returned to grow at 28/20°C. All collected data (mean ± standard deviation SD with 6 replicates) were presented as relative values to the maximal quantum yield of photosystem II photochemistry (Fv/Fm ratio) at −3 d (100% Fv/Fm ratio = 0.805 for XLZ13 and 0.812 for XLZ33 leaves). The chilling stress pre-treatment period was indicated by time points from −3 to 0, and the period after inoculation was indicated by time points from 0 to 15.

Fv/Fm value, maximum quantum yield of photosystem II (PSII) photochemistry (maximum variable fluorescence/maximum yield of fluorescence), characterizes the maximal quantum yield of the primary photochemical reactions in dark adapted leaves [35]. The chilling stress of 16/12°C and 12/8°C pre-treatments caused significant decrease of Fv/Fm ratio in leaves (Figure 7c, d), which indicated the pronounced impairment in the reaction centers of PSII in energy utilization by chilling treatments. Fv/Fm ratios were gradually increased to normal level within 2–4 days upon removed to optimal temperature, indicating short-term chilling did not cause severe PSII photoinhibition and photosynthesis ability was recovered from the chilling stress pre-treatments impairment (Figure 7c, d: line 2, 4). While these chilling stress pre-treated leaves were further inoculated with *A. alternata*, an apparent temporary increase in Fv/Fm ratios occurred by 2 days after inoculation but this was followed by an sustained irreversible decline in a time depended manner (Figure 7c, d: line 1, 3), suggesting irreversible impairment of PSII reaction centers in these infected leaves.

## Discussion

Premature leaf senescence has developed to be one of the important restricted barriers for cotton production in recent years [10], [12]. Understanding the causes of cotton early leaf senescence would help us to avoid cotton premature leaf senescence through appropriate management.

At present, the attempted investigations of the factors for causing cotton leaf senescence were restricted in two reasons. Firstly, plant leaf senescence was an extremely complex biological process. It was still far from revealing mechanisms of this mystical biological process at physiological and molecular levels. Secondly, even the basic investigations the key onset and progression factors for causing cotton early leaf senescence were too difficult to be conducted in precisely controlled conditions when facing the large cotton plants. So far, the investigation of the factors for causing leaf senescence were mainly conducted in rough field conditions, such as: early fruit removal for regulation of cotton's sink-source relationships to proper niches [36]; relatively late-planted cotton with increased plant density for improving cotton growth and the uptake of potassium [37]; potassium supply for providing the nutritional requirement of cotton [21], [38]; It would be possible for application of external plant hormones such as cytokinin, gibberellin or auxin for controlling cotton premature leaf senescence with the revealing the characteristics of phytohormons changes during cotton senescence process [39]. However, the experiments conducted under rough field condition often showed us the puzzled results and unclear information, which often disturbed us in revealing the true factors for causing cotton premature leaf senescence.

In this work, the relationship between the chilling stress and *A. alternata* infection for causing cotton leaf senescence had been investigated under precisely controlled conditions with four to five leaves stage cotton plants, which acted as feasible experiment materials performed in laboratory conditions. As we known, *A. alternata* which was not previously considered as a primary pathogen in cotton but only a weak pathogen infecting young and senescent cotton plants [40]. The collected results presented in our experiment supported that chilling stress acted as the key predisposing factor for causing Alternaria disease. The presented results were also consistent with previous studies performed on other plant diseases. Such as, it was reported that pre-inoculation temperature at 12.9°C often increased susceptibility in wheat to stripe rust [41]. It was also reported that tobacco callus cultures inoculated with tobacco mosaic virus (TMV) and soybean callus cultures inoculated with southern bean mosaic virus (SBMV) revealed a higher rate of virus synthesis held for 4 days at 10°C than those continuously maintained at 24°C [42].

As results showed, short-term chilling stress could cause a certain degree of physiological impairment in cotton leaves as reflected by increase in MDA contents and electrolyte leakage, decrease in chlorophyll contents, soluble protein contents and Fv/Fm ratio (index for maximum quantum efficiency of photosystem II photochemistry measured by chlorophyll fluorometer), et al. While the presented results also showed that all these various physiological impairments could be recovered to normal level in 2–4 days when the chilling stresses were discarded and cotton plants were maintained at optimal temperature again. It suggested that the single adverse factor of short-term chilling stress was not enough for causing cotton leaf senescence.

When the chilling stress injured cotton leaves were further inoculated with *A. alternata*, the pronounced appearance and development of Alternaria disease, and eventual symptoms of leaf senescence were occurred in sequence. On the one hand, it could be explained that physiological impairment caused by chilling stress, especially the enhancement leakage of ions, nutrition and energy sources into the intercellular spaces could be in favor of the intensified *A. alternata* initial colonization, which may just be the key factor for the weak pathogens infection like *A. alternata* as mentioned [40]. On the other hand, short-term chilling stress would have caused physiological impairment in cotton leaves, including alterations in the homeostasis, stability, biosynthesis and compartmentalization of leaf cells and in decreased fluidity and increased permeability of cellular membranes, which may cause reduction of tissue resistance to Alternaria disease, at last cotton leaves were promoted to the onset of senescence process with the development of Alternaria disease as reflected in irreversible increasing in MDA content and electrolyte leakage, irreversible decreasing in chlorophyll content, soluble protein content, and Fv/Fm ratio. These irreversible changes, especially the lost of primary photochemical reactions as reflected by irreversible decreasing in Fv/Fm ratio indicated that the leaves have got to be senescent [6], [7].

Results of this study showed that *A. alternata* got easier to infect pre-chilling stress cotton leaves, causing severe leaf spot disease, and then accelerating the progression of cotton leaf senescence. The results indicated that leaf spot disease caused by *A. alternata* could become a severe problem for cotton production in certain cold cloudy weather on susceptible cotton cultivars.

Plant leaf senescence induced by *A. alternata* infection had been proved previously. *Alternaria* species were found to produce non-host specific and host-specific toxins [43]. There were at least 12 host-specific toxins produced by plant pathogenic *Alternaria* species and most of species appear to be variants of *A. alternata* that have a distinct and limited host range [44]. It had been suggested that the mode of action of *Alternaria* toxins involves a mechanism of plant cell death [45]. As we know, plant leaf senescence was also considered to be a plant cell death process, which involves the regulated disintegration of cells and tissues that allows for nutrient recycling [46]. Recently, it was reported that metabolic products of *A. alternata* significantly accelerated senescence in the tobacco leaves [47]. Considering of *A. alternata* phytotoxins produced in infected cotton leaves [48], it is quite possible that *A. alternata* infection accelerates the progression of cotton leaf senescence as the function of *A. alternata* toxins. Further studies are expected to explore the mechanism roles of *A. alternata* toxins in inducing senescene.

Compared to XLZ13, XLZ33 was more resistant to cotton premature leaf senescence in our previous field investigation [14]. In this case, the differences in development and severity of Alternaria disease could also be distinguished between XLZ13 and XLZ33. As results showed, compared to XLZ33, there were a delay in appearance of Alternaria disease, relatively lower values of disease index and relatively slight cell impairment reflected in various detected physiological indexes in the chilling stress pre-treated and inoculated XLZ13 leaves. But in overall, these XLZ33 leaves still could not be excluded from the infection by *A. alternata* and emergence of severe Alternaria disease and leaf senescence symptoms. It could be explained by the heavy inoculation manipulation or critical chilling stress pre-treatments performed on young cotton leaves.

It would be great helpful for taking appropriate management to control cotton premature leaf senescence with the understanding that cotton leaf senescence was accelerated by *A. alternata* infection and development of Alternaria disease which was promoted by the condition of pre-chilling stress. The following available candidate approaches for alleviating cotton premature leaf senescence could be recommended. Such as, the methods for improving chilling stress resistance of cotton, the methods for prevention of cotton Alternaria disease occurrence and development, and planting leaf premature senescence resistant cotton cultivars. Considering premature leaf senescence occurred in the late cotton growth stage, while our experiments for revealing the relationship of chilling stress, *A. alternata* infection and cotton leaf senescence were conducted in the seedling stage. It should be further investigated under field conditions to confirm whether these recommended approaches are effectivey for controlling cotton premature leaf senescence.

## Materials and Methods

### Plant and fungus materials

Leaf premature senescence resistant cultivar XLZ33 and susceptible cultivar XLZ13, were kindly provided by Li Jia-Sheng, researcher of Institute of Agricultural Sciences, Kuitun, Xinjiang. Premature Leaf senescence characters for two upland cotton (*Gossypium hirsutum* L.) cultivars were further confirmed by our previous study [14]. The cotton seeds were surface-sterilized for 2 min in 75% (v/v) ethanol and 10 min in 6% (v/v) sodium hypochloride solution, then thoroughly washed with sterilized water, and germinated on two sheets of moist filter paper at 28°C in a growth chamber, then transferred to 10-cm diameter plastic pots filled with a mixture of field soil: commercial humus: commercial vermiculite (1∶1∶1, v/v/v) and planted in a greenhouse with a natural photoperiod (day: 25–30°C, night: 20–25°C) in May/June. The planted cotton were periodically irrigated and fertilized until used for experiments.

The identified *A. alternata* isolate A1 used in this study was isolated from cotton leaf spot disease lesions and maintained in our laboratory on potato-dextrose agar (PDA) slants at 4°C [25].

### Chilling stress pre-treatments of cotton plants

Two kinds of chilling stress pre-treatment were performed on four-to-five leaves stage cotton plants in controlled environment chambers (Growth Cabinet MLR-350, Sanyo, Osaka). One pre-treatment experiment was conducted at series of low temperatures of 20/16°C, 16/12°C, 12/8°C day/night for a fixed time length of 3 days. The other was conducted at the fixed low temperature regime of 16/12°C day/night with various durations. The control plants were sustained growing at optimal temperature of 28/20°C day/night. All experiment chambers were set with a 14/10 hours photoperiod at 300 µmol photons m^−2^ s^−1^ and a relative humidity of 65%. Each treatment was replicated at least three times and plants positions within the chamber were random and changed daily.

### *A. alternata* inoculation procedure

The inoculum of *A. alternata* isolate A1 was prepared as previously described [49]. 14-day-old cultures grown on PDA slants at 26 to 28°C were scraped with a sterile inoculating loop, and then suspended with sterile deionized water. The suspension was further filtered through cheesecloth, diluted to optimal concentration of 1.2×10^4^ conidial/mL, and then used for inoculation. Spore concentration was estimated microscopically with a counting chamber (Büker type).

The third and forth true leaves of 20 seedlings from each chilling stress treatments were inoculated with the freshly prepared conidial suspension, while mocked control leaves were treated with sterilized water. Inoculation was accomplished by slightly brushing the leaves with a small brush about 1 mL spore suspension per leaf. Subsequently inoculated leaves were covered with a separate, loosely sealed, pre-wetted polyethylene bags. After inoculation, both inoculated and mocked control plants were incubated in a controlled growth chamber in the dark for 24 h at 28±2°C [50]. Then all plants were transferred and maintained at optimal temperature of 28/20°C day/night and with the photoperiod and humidity condition as described above. All treatments were arranged in completely randomized design with three replications.

### Alternaria disease evaluation

After inoculation, each inoculated leaf was inspected daily for the appearance of spot disease symptoms. For evaluation of Alternaria disease, diseased leaves were photographed at 15 days after inoculation with a standard digital camera. The collected digital images were further performed for the analysis of affected leaf area with software Image-Pro Plus (Image-Pro Plus, Version 6.0, Media Cybernetics, L. P, Silver Spring, MD) [51]. The degree of Alternaria disease was divided into six grades with disease scores ranging from 0 to 5 based on the percentage of affected leaf area as previously described [52] with slight modifications: 0 = no disease; 1 = minute pinhead size spots, less than 1% leaf tissue diseased; 2 = small brown to dark brown necrotic lesions, 1 to 5% diseased; 3 = necrotic lesions coalescing, 5 to 10% diseased; 4 = necrosis lesions coalescing, 10 to 20% diseased; and 5 = lesions coalescing, >20% diseased, and/or with desiccated and abscised leaves. The disease index was calculated according to the following formula:
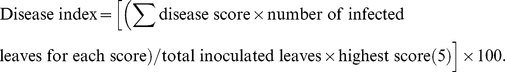



### Biochemical assays

All measurements introduced below were measured at −3 (before chilling stress treatment), 0 (just after chilling stress treatment and before inoculation), 2, 4, 6, 10, 15 days after inoculation, respectively. All measurements were made on the third of fourth fully expanded leaves at 25°C. And the measured leaf areas were selected about 1–2 cm beyond from the edge of the disease spot on diseased leaves unless otherwise noted.

### Membrane electrolyte leakage determination

Membrane electrolyte leakage was determined by measuring electrolyte ion leaked from leaves as described previously [53]. Briefly, 10–15 leaf discs excluding the main veins were washed and placed in 20 mL of deionized water. Water conductivity was recorded at the beginning of the incubation (initial cond.) and after incubation for 3 hours with gentle shaking (cond. 3 h), at 25°C. Then leaf discs were boiled for 5 min for the determination of maximum conductivity (max. cond.). Electrolyte leakage was calculated as: [(cond. 3 h−initial cond.)/(max. cond.−initial cond.)]×100.

### Malondialdehyde (MDA) and soluble protein measurements

Leaves segments were collected as an average sample from at least 10 leaves and immediately frozen in liquid nitrogen and stored at −80°C until used for MDA or soluble protein measurements.

The MDA content was determined according to methods primarily described by Peever and Higgins [54], and modified by Lv et al [55]. Cotton leaves were homogenized in 5 ml of 10% (w/v) trichloroacetic acid (TCA), and centrifuged at 12,000×g for 10 min. A volume of 2 ml of clear supernatant was added to 4 ml of 0.6% (w/v) thiobarbituric acid (TBA, in 10% TCA) and the reaction mixture was incubated at 100°C in water bath for 15 min. The reaction was terminated at room temperature, and the absorbance of the supernatant at 450, 532 and 600 nm was determined with a spectrometer. The concentration of MDA was calculated by the formula: C (µmol/L) = 6.45 (OD_532_−OD_600_)−0.56OD_450_.

Total soluble protein was determined according to the Bradford method [56] with bovine serum albumin as the standard.

### Measurement of chlorophyll content

Relative chlorophyll content per unit leaf area was determined using a portable chlorophyll SPAD-502 (Minolta Crop., Osaka, Japan) which measures transmission of intact leaves at two wavelengths of 650 and 940 nm, and the measuring area was 6 mm^2^ [57]. Calibrations show that relative SPAD values depend on chlorophyll content in a linear manner over a wide range. Each data point represents the mean value of ten independent measurements.

### Measurements of photosystem II efficiency

Chlorophyll fluorescence measurements after dark adaptation were carried out as described by [58] by a chlorophyll fluorometer (Junior PAM, Walz, Effeltrich, Germany). Mean values of the maximum quantum efficiency of photosystem II photochemistry (Fv/Fm ratio) were based on ten independent measurements.

### Experimental design and statistical analysis

Experiments were carried out in a completely randomized design with three replicates for each treatment. All experiments were repeated twice and data from each repeat experiment were analyzed separately and in combination. Results of the two repeats analyzed separately and in combination had similar responses. So data from the two repeats were combined (six replicates) and analyzed together using the SAS program (SAS Institute, Cary, NC, USA).

All data (mean ± standard deviation (SD)) except for electrolyte leakage values were presented relatively to their values at −3 d ( = 100%, before chilling stress pre-treatments) for better readability of the figures and to stress their changes in time. Differences were considered significant at a probability level of *P*<0.05.
